# Phylogenetic and distributional data on boletoid fungi (Boletaceae) in Cyprus and description of a new sampling methodology

**DOI:** 10.1016/j.dib.2019.104115

**Published:** 2019-06-11

**Authors:** Michael Loizides, Jean-Michel Bellanger, Boris Assyov, Pierre-Arthur Moreau, Franck Richard

**Affiliations:** a P.O. Box 58499, Limassol, 3734, Cyprus; b UMR 5175 CEFE, Université de Montpellier, INSERM, Campus CNRS, 1919 Route de Mende, 34293, Montpellier, France; c Institute of Biodiversity and Ecosystem Research, Bulgarian Academy of Sciences, 2 Gagarin St, 1113, Sofia, Bulgaria; d Faculté de Pharmacie Lille, Université de Lille, EA 4483 IMPECS, F-59000, Lille, France

**Keywords:** Boletales, Boletus, Fungi, Island biogeography, Inventory, Mediterranean

## Abstract

The data presented here was obtained during a decade-long macromycete inventory on the island of Cyprus and is supplementary to the research article “Present status and future of boletoid fungi (*Boletaceae*) on the island of Cyprus: cryptic and threatened diversity unravelled by ten-year study” [1]. A new, rainfall-based sampling protocol for documenting fungal diversity in Mediterranean ecosystems, is described in detail.

**Table:** 

Subject area	*Mycology, Ecology, Biological Conservation*
More specific subject area	*Molecular phylogenetics, Systematics, Island biogeography, Climate change*
Type of data	*Table, figure*
How data was acquired	*Field data was obtained through surveying and sampling during a 10-y-period (2007–2016); field photos were taken with a CANON EOS DIGITAL camera; Microscopic studies were performed under a Leica BM E binocular, an AmScope T360B trinocular, and a Zeiss axioskop microscopes; DNA extraction and PCR amplification were conducted with the REDExtract-N-Amp; multiple sequence alignment was carried out with MUSCLE 3.7; precipitation data was retrieved from the Cyprus Department of Meteorology; distribution maps were compiled in QuarkXPress 14.2.1 based on records reported in* Ref. [1].
Data format	*Raw and Analyzed*
Experimental factors	*Data was obtained during a 10-year-long inventory on the island of Cyprus, situated 35.1264° north and 33.4299° east in the Mediterranean basin and occupying an area of 9,*251km*.*
Experimental features	*Thirty sites representative of all major ectomycorrhizal (EcM) habitats on the island were preselected and systematically surveyed following rainfall episodes, as part of a general inventory carried out on the island (see*Table 1, Table 2*and*Fig. 1*). All data belonging to the Boletaceae family was then extracted from the general inventory data and analyzed separately.*
Data source location	*Cyprus*
Data accessibility	*Data is provided in this article*
Related research article	*Loizides M, Bellanger J-M, Assyov B, Moreau P-A, Richard F. (2019) Present status and future of boletoid fungi (Boletaceae) on the island of Cyprus: cryptic and threatened diversity unravelled by 10-year study. Fungal Ecology (in press)*[1].

**Table:** 

**Value of the data** • The rainfall-based sampling methodology followed in this 10-year-long inventory, allowed for the coverage of a very large area across an extended altitudinal gradient and provided excellent yields of fungal diversity, including large numbers of previously undocumented and rare species • The gene flow detected between the two subclades of *Butyriboletus fechtneri sensu lato* helps clarifying the phylogenetic boundaries of this taxon and can be used to determine which infraspecific rank, if any, should be assigned to genetically variable populations • Distributional maps of presently known diversity of *Boletaceae* fungi can be useful for future research and conservation efforts on a local or regional scale

## 1 Data

**Fig. 1:**
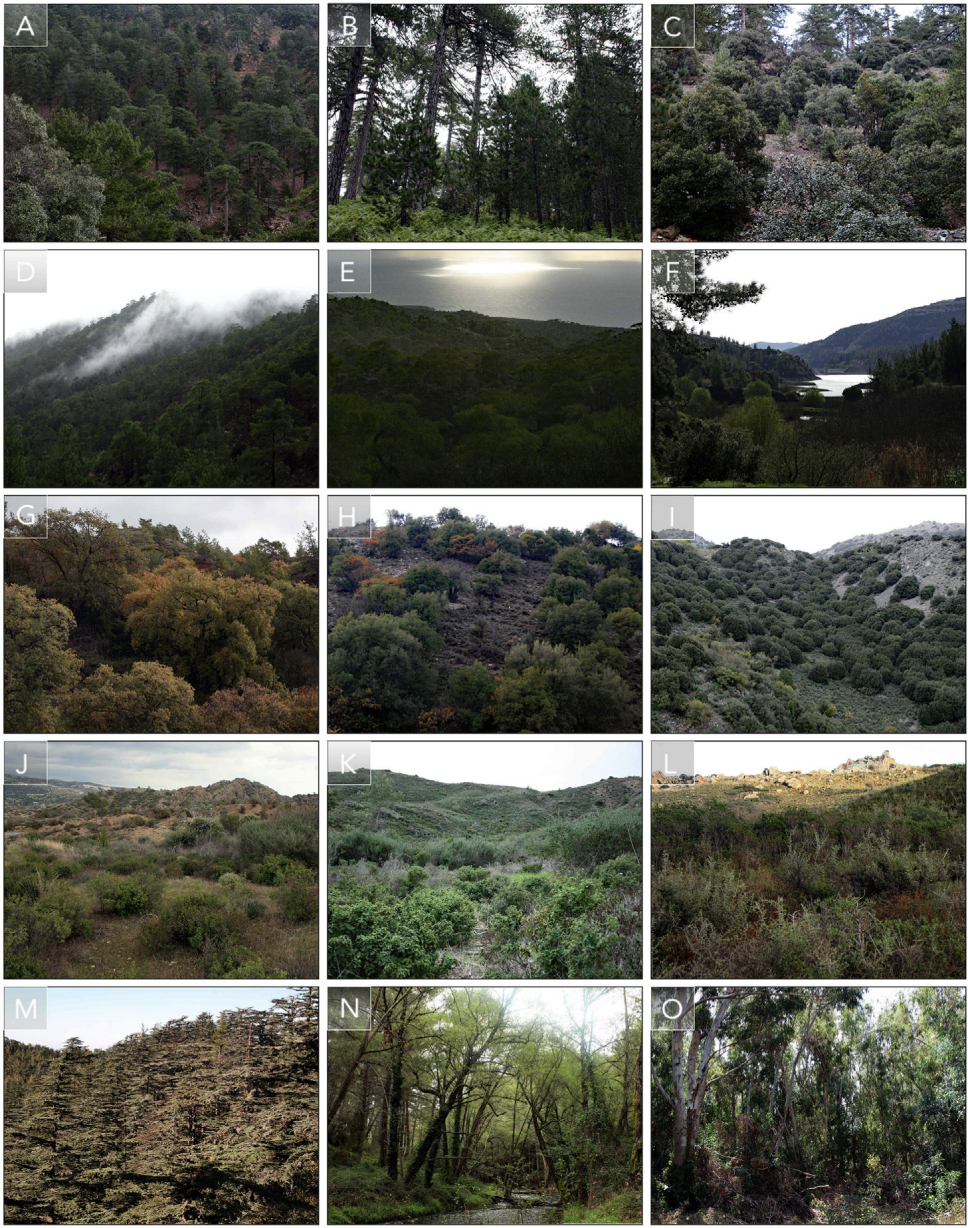
Selection of representative habitats dominated by ectomycorrhizal (EcM) trees and shrubs: (A) *Pinus nigra* supsp. *pallasiana* forest in the oromediterranean belt (1850 m a.s.l.) at Chionistra; (B) *Pinus nigra* supsp. *pallasiana* forest in the supramediterranean belt (1600 m a.s.l.) at Troodos; (C) *Pinus nigra* supsp. *pallasiana* and *Quercus alnifolia* forest in the oromediterranean belt (1750 m a.s.l.), at Troodos; (D) *Pinus brutia* forest in the mesomediterranean belt (1000 m a.s.l.), at Stavros tis Psokas; (E) *Pinus brutia* forest in the thermomediterranean belt (200 m a.s.l.) at Akamas; (F) *Pinus brutia* and *Quercus coccifera* subsp. *calliprinos* forest in the thermomediterranean belt (450 m a.s.l.) at Kelefos; (G) *Quercus infectoria* subsp. *veneris* stand in the thermomediterranean belt (500 m a.s.l.), at Ayia Paraskevi; (H) *Quercus coccifera* subsp. *calliprinos* stand in the thermomediterranean belt (400 m a.s.l.), at Arminou; (I) *Quercus alnifolia* matorral in the mesomediterranean belt (800 m a.s.l.), at Palaichori; (J) *Cistus salvifolius* and *C. cretecus* matorral in the thermomediterranean belt (200 m a.s.l.), at Kalavasos; (K) *Cistus salvifolius* matorral in the thermomediterranean belt (200 m a.s.l.), at Asgata; (L) *Cistus monspelliensis* matorral in the thermomediterranean belt (400 m a.s.l.), at Akamas; (M) *Cedrus brevifolia* forest in the mesomediterranean belt (1200 m a.s.l.), at Tripilos; (N) Riparian *Alnus orientalis* forest in the thermomediterranean belt (400 m a.s.l.), at Kelefos; (O) *Eucalyptus camaldulensis* and *E. gomphocephala* plantation in the dunal belt (5 m a.s.l.), at Fassouri.

**Fig. 2:**
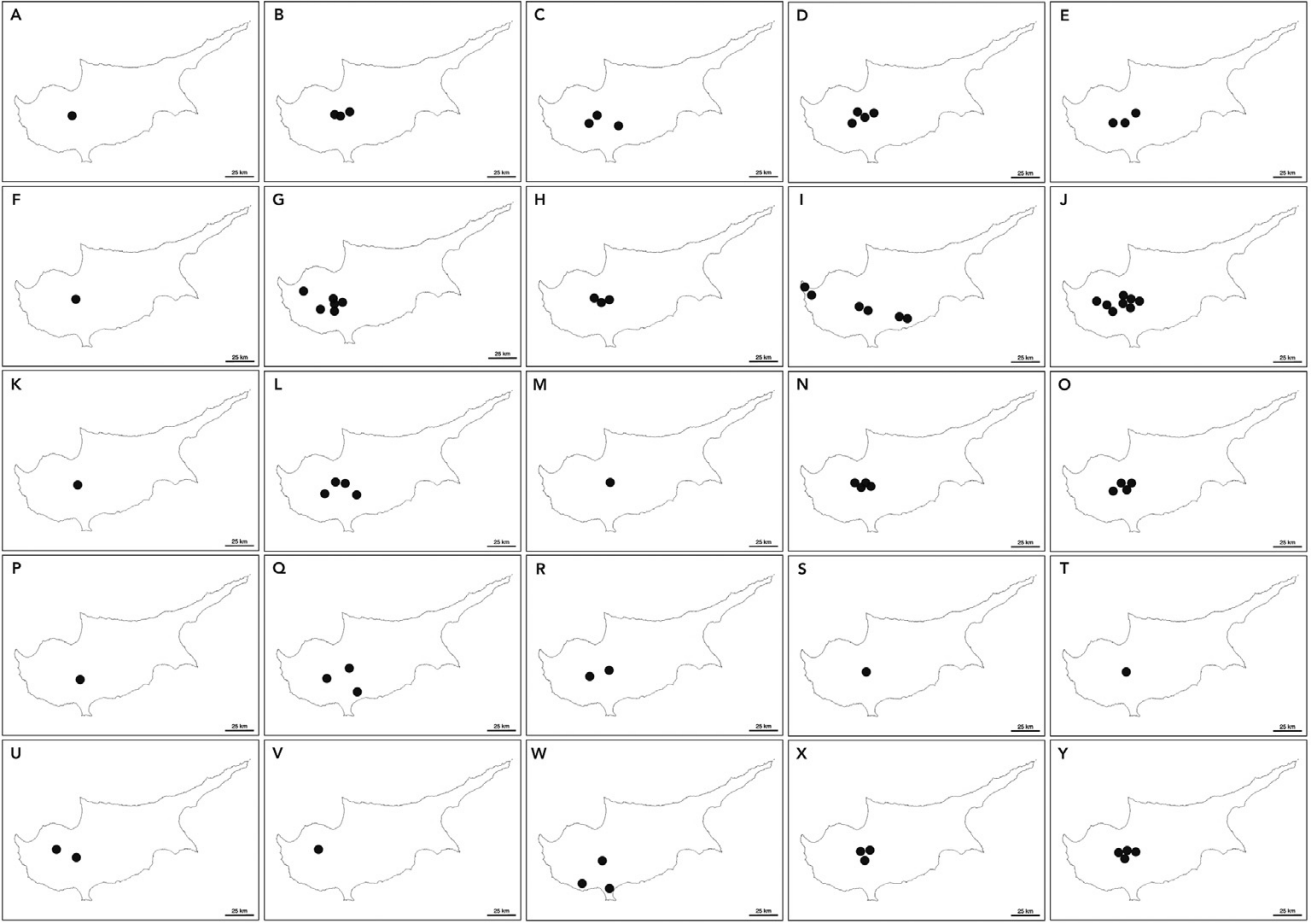
Distribution of *Boletaceae* fungi in Cyprus based on a systematic assessment of field collections: (A) *Alessioporus ichnusanus*; (B) *Boletus aereus*; (C) *Butyriboletus fechtneri s.l*; (D) *Caloboletus radicans s.l*; (E) *Chalciporus amarellus*; (F) *Exsudoporus permagnificus*; (G) *Hemileccinum impolitum*; (H) *Imperator luteocupreus*; (I) *Leccinellum corsicum;* (J) *Leccinellum lepidum;* (K) *Rheubarbariboletus persicolor aff;* (L) *Rubroboletus lupinus s.l;* (M) *Rubroboletus pulchrotinctus;* (N) *Rubroboletus rhodoxanthus;* (O) *Rubroboletus satanas;* (P) *Suillellus adonis;* (Q) *Suillellus comptus;* (R) *Suillellus luridus;* (S) *Suillellus mendax;* (T) *Suillellus queletii;* (U) *Xerocomellus chrysenteron;* (V) *Xerocomellus cisalpinus;* (W) *Xerocomellus redeuilhii;* (X) *Xerocomellus sarnarii;* (Y) *Xerocomus subtomentosus*.

The high-profile family of *Boletaceae* accommodates rare as well as economically important terrestrial fungi with tubular hymenophores and dark, usually fusiform or subfusiform spores [2], [3], [4]. Although the family has been intensively studied in recent years and extensive systematic re-arrangements have been proposed, boletoid fungi in Mediterranean and inslular ecosystems remain poorly documented. The data presented here is supplementary to the research paper “Present status and future of boletoid fungi (Boletaceae) on the island of Cyprus: cryptic and threatened diversity unravelled by ten-year study” [1], and was obtained during a 10-year macromycete inventory on the Mediterranean island of Cyprus. A new, rainfall-based sampling methodology is introduced and described in detail, designed to produce maximum yields of fungal diversity in Mediterranean ecosystems, where rainfall is unpredictable and uneven in distribution, and fungal fruitings are consequently prolific but localized and brief (Table 3). Thirty representative sites dominated by ectomycorrhizal trees and shrubs, were preselected and systematically surveyed, following rainfall episodes (Table 1, Table 2, Fig. 1). In addition, the known distribution of boletoid species documented on the island is depicted (Fig. 2), and the ITS polymorphism within the /*Butyriboletus fechtneri sensu lato* clade is demonstrated (Table 4).

**Table 1:** Permanent sites: Thirty sites pre-selected and regularly surveyed between seasons 2007/08 and 2016/17, including approximate area (m^2^) for each site, elevation, ectomycorrhizal tree composition (habitat), elevation and number of visits per season between 2007/08 and 2016/17.

Locality	District	Area m^2^	Elevation	Habitat (EcM)	07/08	08/09	09/10	10/11	11/12	12/13	13/14	14/15	15/16	16/17	Av.
Troodos East (Makria Kontaria)	Nicosia	∼1km^2^	1600–1750 m	*Pinus nigra ssp. pallasiana*, *Quercus alnifolia*, *Arbutus andrachne*, *Cistus creticus*	4	5	2	1	2	1	1	6	1	1	**2.4**
Troodos Central(Kaledonia trail/Proedriko)	Nicosia	∼2km^2^	1600–1700 m	*P. nigra* ssp. *pallasiana*, *Q. alnifolia*, *Alnus orientalis*, *A. andrachne*, *C. creticus*	7	8	8	1	4	5	3	6	3	1	**4.6**
Troodos Central (Kyvernitikes katoikies 1)	Nicosia	∼1km^2^	1700–1750 m	*P. nigra* ssp. *pallasiana*	13	19	9	1	8	4	7	7	5	2	**7.5**
Troodos Central (Kyvernitikes katoikies 2)	Nicosia	∼1km^2^	1600–1700 m	*P. nigra* ssp. *pallasiana*, *Q. alnifolia*, *A. andrachne*, *C. creticus*	8	16	7	1	8	5	7	9	6	3	**7**
Troodos South (Kataskoinoseis)	Nicosia	∼2km^2^	1400–1600 m	*P. nigra* ssp. *pallasiana*, *Q. alnifolia*, *A. andrachne*, *C. creticus*	7	6	3	1	4	3	2	5	1	2	**3.4**
Troodos North (Almyrolivado/Livadi Pasia)	Nicosia	∼2km^2^	1600–1700 m	*P. nigra* ssp. *pallasiana*, *Cedrus brevifolia*, *Q. alnifolia*	3	4	3	1	3	4	4	3	1	1	**2.7**
Prodromos South (Kampos tou Kalogirou)	Nicosia	∼700m^2^	1300–1400 m	*P. nigra* ssp. *pallasiana*, *Q. alnifolia*, *A. andrachne*, *C. creticus*	1	1	5	1	2	2	3	1	1	3	**2**
Prodromos North (Prodromos dam)	Nicosia	∼1km^2^	1400–1500 m	*P. nigra* ssp. *pallasiana*, *C. creticus*	1	1	3	1	4	2	2	1	1	2	**1.8**
Platania/Karvounas	Nicosia	∼2 km	1000–1100 m	*P. brutia*, *A. andrachne*, *Q. alnifolia*, *C. salviifolius*, *C. creticus*	2	5	10	1	12	9	4	8	5	1	**5.7**
Amiantos	Limassol	∼500m^2^	1200–1400 m	*P. brutia*, *Q. alnifolia*, *A. andrachne*, *Quercus infectoria* ssp. *veneris*, *Cistus* spp.	1	3	3	1	3	1	2	2	1	2	**1.9**
Trooditissa	Limassol	∼2km^2^	1300–1400 m	*P. nigra* ssp. *pallasiana*, *Q. alnifolia*, *A. andrachne*, *C. creticus*	2	1	2	2	6	4	7	1	4	6	**3.5**
Platres/Caledonian Falls	Limassol	∼2km^2^	1200–1300 m	*P. brutia*, *Q. alnifolia*, *A. andrachne*, *C. salviifolius*, *C. creticus*	9	4	5	4	7	6	2	5	5	7	**5.4**
Moniatis/Platres	Limassol	∼2km^2^	800–900 m	*A. orientalis*, *Q. alnifolia*, *P. brutia*	7	11	3	5	3	7	4	1	1	1	**4.3**
Saittas/Moniatis	Limassol	∼2km^2^	600–750 m	*P. brutia*, *Quercus coccifera* ssp. *calliprinos*, *C. salviifolius*, *C. creticus*	11	5	5	4	5	3	5	2	2	4	**4.6**
Pera Pedi/Mandria	Limassol	∼2km^2^	750–850 m	*P. brutia*, *Quercus infectoria* ssp. *veneris*, *Q. coccifera* ssp. *calliprinos*, *Cistus* spp.	8	15	10	8	5	4	3	3	2	2	**6**
Trimiklini	Limassol	∼500m^2^	600–700 m	*P. brutia*, *Q. coccifera* ssp. *calliprinos*, *C. salviifolius*, *C. creticus*	7	8	4	4	5	4	5	2	2	2	**4.3**
Mesa Potamos	Limassol	∼2km^2^	750–1000 m	*P. brutia*, *Q. alnifolia*, *Q. infectoria* ssp. *veneris*, *A. andrachne*, *Cistus* spp.	4	1	1	2	1	1	4	1	2	2	**1.8**
Ayia Paraskevi	Limassol	∼2km^2^	550–700 m	*P. brutia*, *Q. infectoria* ssp. *veneris*, *Q. coccifera* ssp. *calliprinos*, *A. andrachne*, *Cistus* spp.	1	2	5	7	3	2	2	2	2	3	**2.9**
Germasogeia	Limassol	∼500m^2^	120–150 m	*Q. coccifera* ssp. *calliprinos*, *C. creticus*, *Q. salviifolius*, *C. parviflorus*	2	2	1	2	2	2	1	1	1	1	**1.6**
Asgata	Limassol	∼500m^2^	150–200 m	*C. salviifolius*, *C. creticus*	2	3	2	7	4	1	2	1	1	1	**2.4**
Kalavasos	Limassol	∼1km^2^	150–200 m	*C. salviifolius*, *C. creticus*	1	1	1	3	2	1	2	1	2	1	**1.5**
Pissouri	Limassol	∼800m^2^	200–250 m	*P. brutia*	3	3	2	4	1	2	2	3	1	1	**2.2**
Alassa	Limassol	∼500m^2^	400–450 m	*Salix alba*	1	4	1	2	2	2	4	1	1	1	**1.9**
Akrotiri	Limassol	∼2km^2^	0–10 m	*Pinus halepensis*, *P. brutia*, *Cistus parviflorus*, *C. creticus*, *C. salviifolius*	1	5	1	2	13	4	5	8	3	2	**4.4**
Fassouri	Limassol	∼500m^2^	0–5 m	*Eucalyptus gomphocephala*, *E. camaldulensis*	2	6	3	3	3	3	4	1	1	1	**2.7**
Agios Nikolaos South (Arminou dam)	Paphos	∼1km^2^	450–600 m	*P. brutia*, *Q. coccifera* ssp. *calliprinos*, *C. creticus*, *C. salviifolius*	3	4	6	6	2	8	3	4	1	2	**3.9**
Agios Nikolaos North (Kelefos bridge)	Paphos	∼1km^2^	450–500 m	*P. brutia*, *Q. coccifera* ssp. *calliprinos*, *A. orientalis*, *C. creticus*, *C. salviifolius*	3	4	6	6	1	3	3	4	1	1	**3.2**
Akamas	Paphos	∼2km^2^	100–250 m	*P. brutia*, *C. monspeliensis*, *C. salviifolius*	–	1	1	1	1	–	–	3	1	2	**1**
Cedar Valley	Paphos	∼1km^2^	1000–1200 m	*Cedrus brevifolia*, *P. brutia*, *A. andrachne*, *Q. alnifolia*	1	1	1	3	2	–	–	1	1	–	**1**
Stavros tis Psokas/Kannaviou	Paphos	∼2km^2^	1000–1200 m	*P. brutia*, *A. andrachne*, *Q. alnifolia*, *Q. infectoria* ssp. *veneris*, *Cistus* spp.	1	1	3	2	2	–	–	–	1	1	**1.1**

**Table 2:** Number of surveys per season: Total number of surveys carried out per season between 2007/08 and 2016/17.

	August	September	October	November	December	January	February	March	April	May	June	July	Total no. of surveys
2007–08	3	7	10	12	18	6	4	10	3	–	–	3	**76**
2008–09	3	12	23	15	8	16	21	16	10	4	–	1	**129**
2009–10	–	14	11	14	18	8	10	13	3	–	1	2	**94**
2010–11	3	–	2	6	8	18	15	21	11	3	–	1	**88**
2011–12	–	2	13	13	13	16	18	17	4	2	1	4	**103**
2012–13	4	2	13	14	12	10	8	4	1	1	1	–	**70**
2013–14	–	2	2	6	7	7	12	7	1	4	7	–	**55**
2014–15	4	1	4	9	10	10	8	7	12	–	–	–	**65**
2015–16	–	–	1	8	5	5	7	8	4	1	3	–	**42**
2016–17	–	5	4	1	4	8	–	9	12	2	–	–	**45**
TOTAL	**17**	**45**	**83**	**98**	**103**	**104**	**103**	**112**	**61**	**17**	**13**	**11**	**767**

**Table 3:** Climatological data: Precipitation records per month between 2007 and 2016, based on official data retrieved from the Cyprus Department of Meteorology. Column A: indicates the month for the studied period; Column B: indicates the rainfall average during the 30-years long period 1961–1990, considered as ‘normal’ in the current meteorological database; Column C: indicates the measured rainfall for the considered month of the survey; Column D: indicates the ratio between the two previous columns, demonstrating deficit/excess of rainfall between the observed data and the expected (averages).

Month	Normal (mm)	Actual (mm)	Actual/normal
October 2007	32.70	10.20	0.31
November 2007	53.30	39.90	0.75
December 2007	105.60	90.00	0.85
January 2008	102.40	38.30	0.37
February 2008	81.60	35.60	0.44
March 2008	61.90	21.50	0.35
April 2008	29.90	2.30	0.08
May 2008	19.60	9.50	0.48
June 2008	6.00	0.20	0.03
July 2008	2.60	0.20	0.08
August 2008	2.90	2.20	0.76
September 2008	4.50	22.40	4.98
October 2008	32.70	22.70	0.69
November 2008	53.30	21.50	0.40
December 2008	105.60	95.40	0.90
January 2009	102.40	108.80	1.06
February 2009	81.60	106.30	1.30
March 2009	61.90	75.00	1.21
April 2009	29.90	22.60	0.76
May 2009	19.60	29.20	1.49
June 2009	6.00	0.50	0.08
July 2009	2.60	1.60	0.62
August 2009	2.90	6.10	2.10
September 2009	4.50	37.80	8.40
October 2009	32.70	40.30	1.23
November 2009	53.30	44.50	0.83
December 2009	105.60	152.10	1.44
January 2010	102.40	149.50	1.46
February 2010	81.60	107.50	1.32
March 2010	61.90	2.60	0.04
April 2010	29.90	20.20	0.68
May 2010	19.60	14.10	0.72
June 2010	6.00	9.20	1.53
July 2010	2.60	5.40	2.08
August 2010	2.90	0.00	0.00
September 2010	4.50	1.00	0.22
October 2010	32.70	9.20	0.28
November 2010	53.30	0.10	0.00
December 2010	105.60	109.90	1.04
January 2011	102.40	105.90	1.03
February 2011	81.60	73.60	0.90
March 2011	61.90	68.80	1.11
April 2011	29.90	42.80	1.43
May 2011	19.60	24.30	1.24
June 2011	6.00	9.50	1.58
July 2011	2.60	0.00	0.00
August 2011	2.90	0.60	0.21
September 2011	4.50	20.20	4.49
October 2011	32.70	14.50	0.44
November 2011	53.30	80.50	1.51
December 2011	105.60	117.20	1.11
January 2012	102.40	238.40	2.33
February 2012	81.60	99.40	1.22
March 2012	61.90	39.20	0.63
April 2012	29.90	18.60	0.62
May 2012	19.60	30.60	1.56
June 2012	6.00	9.00	1.50
July 2012	2.60	4.00	1.54
August 2012	2.90	2.90	1.00
September 2012	4.50	0.20	0.04
October 2012	32.70	53.40	1.63
November 2012	53.30	84.40	1.58
December 2012	105.60	209.40	1.98
January 2013	102.40	59.20	0.58
February 2013	81.60	41.70	0.51
March 2013	61.90	11.80	0.19
April 2013	29.90	48.50	1.62
May 2013	19.60	27.10	1.38
June 2013	6.00	0.00	0.00
July 2013	2.60	0.40	0.15
August 2013	2.90	0.00	0.00
September 2013	4.50	7.00	1.56
October 2013	32.70	16.10	0.49
November 2013	53.30	25.10	0.47
December 2013	105.60	58.00	0.55
January 2014	102.40	36.90	0.36
February 2014	81.60	41.50	0.51
March 2014	61.90	27.20	0.44
April 2014	29.90	13.10	0.44
May 2014	19.60	62.90	3.21
June 2014	6.00	15.50	2.58
July 2014	2.60	3.70	1.42
August 2014	2.90	4.50	1.55
September 2014	4.50	10.80	2.40
October 2014	32.70	45.00	1.38
November 2014	53.30	48.30	0.91
December 2014	105.60	84.20	0.80
January 2015	102.40	168.10	1.64
February 2015	81.60	104.60	1.28
March 2015	61.90	62.30	1.01
April 2015	29.90	16.10	0.54
May 2015	19.60	20.10	1.03
June 2015	6.00	5.80	0.97
July 2015	2.60	1.80	0.69
August 2015	2.90	0.90	0.31
September 2015	4.50	4.80	1.07
October 2015	32.70	54.70	1.67
November 2015	53.30	10.20	0.19
December 2015	105.60	34.30	0.32
January 2016	102.40	82.10	0.80
February 2016	81.60	25.80	0.32
March 2016	61.90	49.60	0.80
April 2016	29.90	12.90	0.43
May 2016	19.60	26.80	1.37
June 2016	6.00	3.10	0.52
July 2016	2.60	0.60	0.23
August 2016	2.90	0.90	0.31
September 2016	4.50	8.10	1.80
October 2016	32.70	23.50	0.72
November 2016	53.30	32.30	0.61
December 2016	105.60	163.80	1.55
January 2017	102.40	79.70	0.78
February 2017	81.60	11.80	0.14
March 2017	61.90	59.80	0.97
April 2017	29.90	19.70	0.66
May 2017	19.60	16.30	0.83
June 2017	6.00	2.70	0.45
July 2017	2.60	0.10	0.04
August 2017	2.90	2.40	0.83
September 2017	4.50	1.00	0.22

**Table 4:** (ITS) polymorphisms within *Butyriboletus fechtneri* lineage.

ITS sequence	Position in the alignment																		
	56	76	79	81	89	118	184	207	259	443	461	471	478	550	619	623	693	704	768
FR2015676	A	A	A	–	G	T	C	T	T	–	–	–	–	Y	G	T	–	T	T
FR2015677	A	A	A	–	G	T	C	T	T	–	–	–	–	Y	G	T	–	T	T
FR2016688	A	A	A	–	G	T	C	Y	T	–	–	–	G	C	G	T	–	T	
FR2017042	A	A	A	–	**R**	**Y**	C	T	T	C	–	–	–	C	**R**	T	T	Y	T
FR2017043	A	A	A	–	G	T	C	T	T	–	–	–	–	C	G	T	–	T	T
FR2017060	A	A	A	–	G	T	C	T	T	–	–	–	–	C	G	T	–	T	T
FR2017041	A	A	A	–	G	T	C	C	T	–	A	–	–	C	G	T	–	T	T
KC416637	*A*	*T*	*A*	*G*	*A*	*C*	*C*	*T*	*T*	*C*	–	*T*	–	*C*	*A*	*T*	*T*	–	*T*
HM347652	–	*A*	*G*	*G*	*A*	*C*	*T*	*T*	*C*	*C*	–	–	–	*C*	*A*	*C*	*T*	–	–
KC584784	–	*A*	*A*	*G*	*A*	*C*	*C*	*T*	*T*	*C*	–	–	–	*C*	*A*	*T*	*T*	–	
KJ419929	*A*	*A*	*A*	*G*	*A*	*C*	*C*	*T*	*T*	*C*	–	–	–	*C*	*A*	*T*	*T*	–	*T*
FR2017062	*A*	*A*	*A*	–	*A*	*C*	*C*	*T*	*T*	*C*	–	–	–	*C*	*A*	*T*	*T*	–	
UDB019603	*A*	*A*	*A*	*G*	*A*	*C*	*C*	*T*	*T*	*C*	–	–	–	*C*	*A*	*T*	*T*	–	

Italic represents the sequences resolved in the “pan-European” subclade in Fig. 1D; Bold represents the gene flow-revealing heterozygocities.

## 2 Experimental design, materials and methods

### 2.1 Data collection and sampling methodology

Data on fungi belonging to the *Boletaceae* family was gathered during a decade-long macromycete inventory on the island of Cyprus, between 2006 and 2017, following a modified protocol based on Richard et al. (2004) [5]. Thirty loosely delimited sites were pre-selected and regularly surveyed (see Table 1), in addition to other less frequently visited localities. Fungal diversity was for the most part undocumented on the island prior to this inventory, therefore the sampling strategy was designed to cover as large an area as possible as possible and yield the maximum possible number of species. As a result, pre-selected sites were consequently large, ranging in size from ∼500m^2^ to ∼2km^2^. Selection of the permanent sites included all major habitat types formed by ectomycorrhizal (EcM) trees and shrubs on the island, and preliminary observations, anecdotal reports, altitudinal range, accessibility and mean annual precipitation were also taken into consideration. Single-tree communities are rare on Cyprus, therefore the majority of sites were comprised of mixed-tree communities. Of these, mixed *Pinus brutia*/*Quercus alnifolia* habitats are the most widely distributed woodland habitats on the island and as such were better represented among the permanent sites, but mixed *P. brutia*/*Q. coccifera* subsp. *calliprinos*, and *P. nigra* subsp. *pallasiana*/*Q. alnifolia* habitats were also well-represented (see Table 1). Because fungal fruiting episodes in the Mediterranean region are typically prolific but brief, and seasonal rainfall in Cyprus is uneven in distribution and highly unpredictable, surveys within permanent sites systematically followed rainfall episodes. Precipitation data for each locality was retrieved at least three times a week from the Cyprus Department of Meteorology official website http://www.moa.gov.cy/moa/ms/ms.nsf/DMLindex_en/DMLindex_en?OpenDocument, and forays were planned accordingly. Surveying usually spanned between September and April, 18–20 days following the first substantial rainfall of the season (>20 mm) and regularly thereafter, usually 1–2 days following subsequent rainfall episodes, or 2–4 times a week. In a typical season, surveying begun from the higher elevations of the Troodos massif (1,200–1,950 m above sea level) and, as temperatures dropped and precipitation increased, surveys gradually shifted to the lower elevations, where most of the fruiting occurs during the colder winter months. Surveying for spring species followed the opposite pattern, beginning from the lowlands in late winter and gradually extending to the higher elevations, until mid-to late spring. Exceptionally, surveys were also carried out in the summer months, following substantial precipitation (>30 mm) at the higher elevations of the Troodos mountains (>1,400 m a.s.l.), where brief localized fruitings sometimes occurred. Collection of specimens within the permanent sites was mostly opportunistic and followed fructification patterns, though identified hotspots within each site and certain tree-hosts of interest were regularly checked. Surveys usually lasted 2–4 hours on each site, with 1–4 sites visited in each foray. Highly productive seasons with abundant precipitation and prolific fructifications were more intensively surveyed than seasons with low precipitation and poor fructifications. Overall, a total of 767 forays were carried out during the decade, with a minimum of 42 and a maximum of 129 forays taking place annually, averaging 76.7 forays per season (see Table 2). Over this period, more than 3,500 vouchered collections belonging to over 1,200 species were gathered and archived, from which all relevant data to *Boletaceae* fungi was extracted and analyzed separately.

### 2.2 Ecological, morphological, phenological and chorological analyses

Over 200 *Boletaceae* collections were gathered and analyzed during this ten-year-inventory. All specimens were photographed *in situ*, the altitude and soil characteristics were annotated, and the host plant was assigned based on analysis of plant community composition. For collections found in mixed stands, the putative host-plant was assigned based on analysis of the fruiting pattern, spatial distribution of ECM plants, and known host preferences for each species following original descriptions and monographic works [3], [6], [7], [8], [9], [10], [11]. When the precise ectomycorrhizal symbiont was uncertain, no host-plant was assigned. For the purpose of evaluating estimated abundance, collections found >25 m apart from one-another were considered as fruiting from different mycelia following Dahlberg & Stenlid 1994 [12], and Hirose et al. 2004 [13]. Detailed macromorphological observations were made on fresh fruit bodies, when possible from various developmental stages. Oxidation of the context was observed after bruising the hymenium and longitudinally slicing one or more fresh fruit bodies from each collection. Microscopic studies were performed on both fresh and dried material under a Leica BM E binocular, an AmScope T360B trinocular plan achromatic, and a Zeiss axioskop microscopes at ×100, ×400 and ×1000 magnifications. For spore study, normal tap water was used as a mounting medium. A minimum of 30 naturally discharged, normally developed spores were measured from each basidiocarp, after placing fragments of the pileus on a glass slide overnight. When fresh material was not available, naturally discharged spores deposited on the stipe apex were measured. The Me (average length and width), Q (minimum and maximum length/width ratio) and Qm (average length/width ratio) were calculated for each collection, based on methods described by Peintner et al. (2003) [14], and Assyov (2012) [15]. Melzer's solution was used to observe possible amyloidity of the hyphae at the stipe base, following Singer (1965) [6], and Ladurner & Simonini (2003) [10]. Congo red in 10% ammonia (NH_4_OH), lactophenol cotton blue (LPCB), and 5% potassium hydroxide (KOH) were used to highlight the basidia, cystidia and pileipellis. All climatological data cited in this study (including normal, actual and cumulative actual/normal monthly precipitation), was retrieved from Cyprus Department of Meteorology. Correlation between climatological variables (monthly, seasonal and annual precipitation levels), and fruiting abundance of boletoid fungi, was performed using Pearson's product-moment tests in R 3.2.4 (R Core Team 2016). Distribution maps were compiled in QuarkXPress 14.2.1, based on records reported in Loizides et al. (2019) [1].

### 2.3 DNA extraction, amplification and sequencing

Following morphological studies, representative specimens identified to belong to distinct species were selected for molecular analysis. A number of collections from atypical habitats or displaying unusual features were also molecularly analyzed, along with comparative collections from Bulgaria, Croatia, France, Greece and Switzerland. DNA extraction and PCR amplification were conducted with the REDExtract-N-Amp^tm^ Plant PCR Kit (Sigma-Aldrich, St. Louis, MO, USA), following the manufacturer's instructions. The internal transcribed spacers and 5.8S rDNA (ITS) were amplified from each collection, with the ITS-1F/ITS-4b primer pair, as described in Richard et al. (2015) [16]. When no band was detected by agarose-gel electrophoresis analysis, 1 μL of the PCR product was used as template in a second PCR using the ITS1F/ITS4 primer pair [17]. Amplicons were purified and sequenced by Eurofins Genomics, Ebersberg, Germany. Raw sequence data were edited and assembled with Codon Code Aligner 4.1.1 (CodonCode Corp., Centerville, MA, USA), and deposited in Genbank under the accession numbers indicated in Table 1 [1].

### 2.4 Phylogenetic analyses

Phylogenetic analyses were performed online at www.phylogeny.lirmm.fr. Multiple sequence alignment was carried out with MUSCLE 3.7 [18], using full processing mode and 16 iterations. When required, alignments were edited manually or with Gblocks 0.91b, set to lowest stringency in the selection of conserved blocks [19], [20]. Maximum likelihood (ML) phylogenetic analyses were performed with PhyML 3.0 [21], using the GTR + I + Γ model of evolution. Branch support was assessed using the non-parametric, Shimodaira–Hasegawa, version of the approximate likelihood-ratio test (SH-aLRT), implemented in the latest release of PhyML and which ensures high accuracy when SH-aLRT > 0.8 [22], [23]. Phylogenies were built using FigTree 1.4.2 (http://tree.bio.ed.ac.uk/software/figtree/) and edited with Inkscape 0.91 (https://inkscape.org/fr/).

## Conflict of interest

The authors declare that they have no known competing financial interests or personal relationships that could have appeared to influence the work reported in this paper.
